# Metabolic Syndrome in Estonia: Prevalence and Associations with Insulin Resistance

**DOI:** 10.1155/2012/951672

**Published:** 2012-02-25

**Authors:** Triin Eglit, Tarvo Rajasalu, Margus Lember

**Affiliations:** Department of Internal Medicine, University of Tartu, 6 L. Puusepa Street, 51014 Tartu, Estonia

## Abstract

Recently, it has been suggested that metabolic syndrome should be considered a premorbid condition in younger individuals. We evaluated the prevalence of metabolic syndrome in Estonia and compared the characteristic profiles between morbid metabolic syndrome (previously established diabetes, hypertension, or dyslipidaemia) and premorbid metabolic syndrome subgroups. Our study was a cross-sectional, population-based sample of the general population in Estonia aged 20–74 years (*n* = 495). Metabolic syndrome was diagnosed by National Cholesterol Education Program Adult Treatment Panel III criteria. Insulin resistance was estimated using the homeostasis model assessment (HOMA-IR). The crude and weighted prevalence of metabolic syndrome was 27.9% and 25.9%, respectively. Despite being significantly younger, the premorbid subgroup showed similar levels of insulin resistance as the morbid subgroup (mean HOMA-IR ± SD 2.73 ± 1.8 versus 2.97 ± 2.1, *P* = 0.5). The most important attribute of metabolic syndrome is insulin resistance, which already characterises metabolic syndrome in the early stages of its metabolic abnormalities.

## 1. Introduction

The metabolic syndrome (MS) has been characterized by a clustering of multiple metabolic risk factors for cardiovascular disease and diabetes. Such factors include glucose intolerance, central obesity, hypertension, elevated triglycerides, and/or low HDL-cholesterol. There is no generally accepted pathophysiological model of MS, but insulin resistance has been proposed as a central underlying mechanism [1]. 

Individuals affected by MS have at least a fivefold increased risk of type 2 diabetes and a twofold increased risk of cardiovascular disease [2, 3], the latter being independent of classical risk factors, such as high LDL-cholesterol and smoking. Various diagnostic criteria of MS have been proposed by different organisations. Most recent definitions include the National Cholesterol Education Program Adult Treatment Panel III (NCEP ATP III) criteria developed by the American Heart Association/National Heart, Lung, and Blood Institute [4] and the 2005 International Diabetes Foundation (IDF) criteria [5]. In 2009, a Joint Interim Statement of several international organisations agreed on the threshold values of all the main components of MS (with the exception of waist circumference) and declared that 3 out of 5 abnormal findings would qualify an individual for MS [6].

MS has become a worldwide epidemic, and its prevalence continues to rise. Among adults in the United States, the prevalence of the ATP III-defined MS increased from 23.7% in years 1988–1992 to 34.6% in years 1999–2002 [7, 8]. While the prevalence of MS is lower in Northern and Mediterranean Europe, it is increasing in many less developed nations [9]. Estonia is a small country in north-east of Europe, whose population has undergone a rapid transition towards a Western sedentary lifestyle over the last decades. The prevalence of MS has not been previously studied in Estonia.

MS may be present in a wide range of groups, from apparently healthy younger individuals to older individuals with advanced stages of cardiovascular disease. There is an ongoing debate about the practical utility of MS [10–12]. Nevertheless, it has become widely accepted that it is a valuable tool for the early recognition of a high life-time risk of diabetes and CVD [11, 13, 14].

The main aim of our population-based study was to assess the prevalence of MS in Estonians aged 20–74 years. In addition, we identified a subgroup of apparently healthy subjects (without previously established diabetes, hypertension, or dyslipidaemia) among all those with clinical MS, and we explored the characteristic profile of this subgroup in comparison to the morbid MS subgroup.

## 2. Methods

A population-based cross-sectional multicentric study on the prevalence of metabolic disorders and associated risk factors was conducted between November 2008 and May 2009 in three different counties of Estonia. The study population consisted of randomly selected adults aged 20−74 years from four general practitioner (GP) practices. The initial study population was selected to be representative of the general Estonian population in terms of age and gender. An invitation letter about the study was sent to each participant. The total response rate was 53.2% (42.3% in the youngest age-group and 65.9% in those aged 45–74 years), resulting in a total study population of 495 subjects, of which 214 were male and 281 female. This left a slight (<5%) under representation of younger (20–39 years) age groups when compared to the Estonian population. 

On the day of the study, subjects were invited to their GP surgery in the morning between 8 a.m. and 11 a.m. after an overnight fast (lasting at least 10 hours). An informed consent form was signed, and blood pressure, waist circumference, height, and weight were measured with the participants wearing their indoor clothes without shoes. Weight was measured to the nearest 0.1 kg using a mechanical scale. Height was measured to the nearest 0.5 cm using a stadiometer. Waist circumference was measured midway between the lower rib margin and iliac crest. Blood pressure was measured using a mercury sphygmomanometer after the patient had been sitting for at least five minutes. The mean of three consecutive measurements was used for analysis, with at least a three-minute interval between each measurement.

Blood samples were taken to measure fasting glucose, insulin, total cholesterol, HDL-cholesterol, and triglycerides. For all nondiabetic subjects, the oral glucose tolerance test was conducted by standard methodology (WHO 1999) with Glycodyn solution (Biofile Ltd, Turku, Finland). A face-to-face clinical interview was conducted to assess other medical conditions and cardiovascular risk factors.

Plasma glucose was measured by the hexokinase method. Total cholesterol, HDL cholesterol, and triglycerides were measured using an enzymatic colorimetric assay (COBAS INTEGRA 800 plus analyzer, Roche, Basel, Switzerland). Plasma insulin was measured using a chemiluminescent assay (Immulite 2000 analyzer, Siemens Healthcare Diagnostics, Deerfield, IL, USA). 

Metabolic syndrome was diagnosed by having at least three of the following NCEP ATP III criteria [4]: waist circumference ≥102 cm in men and ≥88 cm in women, blood pressure ≥130/85 mmHg or taking antihypertensive medication, fasting glucose: ≥5.6 mmol/L or previously diagnosed diabetes, triglycerides ≥1.7 mmol/L or taking lipid-regulating medication, high-density lipoprotein (HDL) cholesterol <1.03 mmol/L in men and <1.30 mmol/L in women, or drug treatment for reduced HDL cholesterol. For subgroup analysis all subjects with MS were divided into two groups: apparently healthy subjects without previously diagnosed diabetes, dyslipidaemia, or hypertension (premorbid subgroup) and unhealthy subjects with previous diagnosis of any of the aforementioned conditions (morbid subgroup). Diabetes, impaired glucose tolerance, and impaired fasting glucose were diagnosed according to WHO criteria [15]. Insulin resistance (IR) was estimated using the homeostasis model assessment (HOMA) formula: HOMA-IR = fasting glucose (mmol/L) × fasting insulin (mU/L)/22.5. IR was defined as the upper quartile of HOMA-IR in the whole study group (exempting subjects with previously known diabetes mellitus), the threshold of which in whole study group was found to be 1.92 (2.04 and 1.82 for men and women, resp.). The subjects with previously diagnosed diabetes were excluded from the calculation of the quartiles of HOMA-IR for the potential impact of diabetes medication on glucose and insulin values. In the subgroup of subjects with previously diagnosed diabetes, the mean HOMA-IR (±SD) was 3.85 ± 3.13. 

The study was approved by the University of Tartu Ethics Review Committee on Human Research.

### 2.1. Statistical Analysis

Due to the slight under representation of younger age-groups (20–39 years), the prevalence of MS was weighted to the Estonian 20–74 year old population (estimated in 2009). Descriptive analysis methods as means and standard deviations were calculated for the continuous variables. The Man-Whitney *U*-test was used for comparisons between different subgroups. The prevalence of MS was presented as a proportion with 95% confidence intervals (95% CI-s). The chi-square test (with Bonferroni correction) was used for multiple comparison of prevalence between three age-groups. *P* values were considered statistically significant at the 0.05 level. Statistical analysis was performed using *R* software* version *2.10.1.

## 3. Results

The prevalence of MS in our study of Estonian adults was 27.9%, 30.8% in men and 25.6% in women. The prevalence of MS in our population, weighted for the average Estonian population (estimated in 2009), was 25.9%, 29.4% in men and 23.8% in women. The prevalence of MS increased significantly with age (Table 1). Prevalence was significantly (*P*-value = 0.02) higher in men when compared to women in the youngest age-group only, and there were no gender-specific differences in prevalence in the middle and older age-groups (Table 1). Prevalence data on diabetes, impaired glucose tolerance, and impaired fasting glucose in our study population has been published elsewhere [16]. Most of the subjects with MS had 3 components of the syndrome (56.4%), 31.5% had 4, and 12.1% had all 5 components. Arterial hypertension (93.6%), abdominal obesity (91.4%), and impaired glucose metabolism (71.4%) were the most common abnormalities in both sexes. 

Anthropometric and biochemical characteristics of subjects with and without MS are shown in Table 2.

We also compared pre-morbid subjects with MS against morbid subjects with MS in our subgroup analysis. The pre-morbid subgroup was significantly younger and had lower systolic blood-pressure, lower BMI, and smaller waist circumference (for BMI and waist, only the difference between pre-morbid and morbid men reached statistical significance). HOMA-IR values and lipid levels did not differ between morbid and pre-morbid subgroups (Table 3). 

A large difference was observed in the insulin resistance between subjects with MS and without MS: the prevalence of insulin resistance (defined as the top quartile of HOMA-IR in whole study group excluding subjects with previously known diabetes) among subjects with and without MS was 62.3% (95% CI 53.6–70.3) and 12.4% (95% CI 9.2–16.4), respectively. The prevalence of insulin resistance among subjects with MS in the pre-morbid and morbid subgroups did not differ, being 58.8% (95% CI 44.2–72.1%) and 64.4% (95% CI 53.3–74.1%), respectively (*P*-value 0.64).

## 4. Discussion

In this population-based cross-sectional study, we estimated the age- and gender-specific prevalence of MS for 20–74-year-old Estonian adults according to NCEP ATP III criteria. A recent joint scientific statement by different international organisations agreed on the threshold values for most of the components of MS, with the exception of waist circumference for abdominal obesity. The IDF criteria define abdominal obesity at a lower threshold (≥94 cm in men and ≥80 cm in women) than the NCEP ATP III criteria [6]. We chose to use the NCEP ATP III criteria for prevalence estimations, since higher thresholds of waist circumference are generally used in Europe and United States [6]. Moreover, a recent meta-analysis of nearly one million patients convincingly demonstrated a 2-fold increase in cardiovascular outcomes and a 1.5-fold increase in all-cause mortality in individuals diagnosed with MS according to the NCEP ATP III criteria [3], giving further credibility to the practical usefulness of NCEP ATP III criteria.

We believe that our study population is truly representative for the general Estonian population (1.34 million inhabitants in 2009). The sample of 495 subjects represented a mixed urban-rural population of Estonia and it was randomly drawn from the patient registers of four general practice centres. It is important to note that every resident of Estonia is automatically added to a general practitioner register according to their address. In our study, we found 27.9% of subjects to be affected by MS and the weighted prevalence rate of the general population to be 25.9%. However, the low response rate, especially in the youngest age group, may have biased our results because the proportion of healthy subjects may have been higher among those who did not respond. In comparison with the prevalence of MS in other countries, the DECODE study including pooled data from nine European population-based cohorts showed higher average prevalence of MS: 32.2% and 28.5% for men and women, respectively [17]. However, the DECODE study population was notably older (30–89 years). More recent studies from Europe that have estimated prevalence according to NCEP ATP III in age groups comparable to that of our study population have shown comparable results: 25.9% in Norway [18], 28.8% in Turkey [19], and 24.7% in Luxembourg [20]. Furthermore, our estimates of the prevalence of diabetes (7.0%) and impaired glucose tolerance (8.0%) in Estonia are also in comparable to those of other developed countries [16]. Thus, the prevalence of metabolic disorders in Estonia—a former socialist country undergoing a rapid transition to the Western sedentary lifestyle—appears to be similar to the rest of the developed world.

MS was more common in men than women in the younger age group only, followed by an equalisation of the gender-specific prevalence in the middle age group and thereafter. These gender-specific differences in Estonia are generally comparable to the patterns described in other populations [21]. However, the difference in prevalence between the men (25.7%) and women (12.9%) in our youngest age group is more pronounced when compared to data, for example, from the USA [21]. Our neighbouring country Finland recently reported a similar 2-fold increase in the prevalence of MS among 24–39-year-old men, when compared to women [22]. These regional observations are particularly noteworthy given that of all the countries of the European Union, gender differences in life expectancy are also the greatest in the Baltic States [23]. Our findings suggest that the distribution of MS between young men and women may be partly responsible for the large differences in life expectancy in the North-East of Europe, as the life expectancy of such young men may be reduced by subsequent cardiovascular disease and diabetes. Future studies could determine whether young men with MS are so prevalent in North-East of Europe, due to genetic vulnerability, environmental influence, or an interaction between these two.

The practical utility of MS has been greatly challenged during the recent years [10, 11, 24, 25]. The following concerns have been raised by critics of the concept: no unifying pathophysiological mechanism of MS has been identified as yet; the risk of cardiovascular disease conferred by the syndrome appears no greater than the sum of its parts; the rationale for the thresholds of various diagnostic criteria is still poorly defined [24]. Another shortcoming has been the inclusion of individuals with established diabetes and heart disease [11]. However, it is still widely recognised that beyond age, high LDL-cholesterol, and other standard risk factors, MS helps to identify residual vascular risk associated with insulin resistance and atherogenic dyslipidaemia (low HDL-cholesterol, high triglycerides, small dense LDL-cholesterol) [12, 14]. There is a general agreement that MS denotes a high life-time risk of diabetes and cardiovascular disease and it has been proposed that after exclusion of individuals with established diabetes and cardiovascular disease MS should be considered a pre-morbid condition [11]. This prompted us to ask whether in our study population the individuals, who fulfilled the criteria of MS, but had not been previously diagnosed with diabetes, hypertension, and/or dyslipidaemia (high triglycerides and/or low HDL-cholesterol), represented a typical subgroup of subjects with MS as compared with individuals with established cardiometabolic abnormalities. In particular, we were interested whether in the pre-morbid subgroup MS was characterised by insulin resistance. As expected, pre-morbid individuals were significantly younger than morbid subjects, and male subjects were significantly less obese (by both waist circumference and BMI). Despite these differences, pre-morbid subjects proved to be as insulin resistant as morbid subjects estimated both by the mean HOMA-IR and by proportion of the subjects with HOMA-IR in the upper quartile of HOMA-IR in the whole study group. This may indicate that insulin resistance is a hallmark of MS already in the early stage of this disorder. However, insulin resistance of the same magnitude in the premorbid and morbid subgroups with MS should be interpreted with caution, due to the therapeutic effects of lifestyle and medical interventions on alleviating insulin resistance, in the morbid subgroup. Elevations of insulin concentration have been shown to precede the development of diabetes and multiple metabolic disorders in large prospective studies [26, 27]. Moreover, insulin resistance *per se* is a well-established independent predictor of cardiovascular disease [28, 29]. In line with previous studies [30, 31], our results support the conclusion that a high proportion of insulin resistant subjects, both apparently healthy individuals and those with established cardiometabolic abnormalities, can be identified by simple measurements defining MS. Therefore, MS seems to remain a useful practical tool for early identification of apparently healthy individuals at a considerable risk of cardiovascular disease and diabetes.

## 5. Conclusions

The prevalence of metabolic syndrome (as defined by NCEP ATP III) in Estonia was comparable to other European countries. However, it appears that younger men in Estonia have a relatively high prevalence of MS. Insulin resistance already characterised MS in apparently healthy individuals without previously diagnosed comorbidities. This suggests that the concept of MS may be practically useful in the early prediction of life-time risk of cardiovascular disease and diabetes.

## Figures and Tables

**Table 1:** Age- and gender-specific prevalence of metabolic syndrome in Estonia.

Age–group	*n*	% (95% CI)
20–44 years (*n* = 221)	42	19.0 (14.2–24.9)
Men (*n* = 105)	27	25.7 (17.9–35.3)
Women (*n* = 116)	15	12.9 (7.7–20.7)
45–60 years (*n* = 163)	46	28.2 (21.6–35.9)
Men (*n* = 64)	19	29.7 (19.2–42.6)
Women (*n* = 99)	27	27.3 (19.0–37.3)
61–74 years (*n* = 111)	50	45.0 (35.7–54.8)
Men (*n* = 45)	20	44.4 (30.0–59.9)
Women (*n* = 66)	30	45.5 (33.3–58.1)
Total (*n* = 495)	138	27.9 (24.0–32.1)
Men (*n* = 214)	66	30.8 (24.8–37.6)
Women (*n* = 281)	72	25.6 (20.7–31.2)

**Table 2:** Comparison of subjects with and without metabolic syndrome (MS).

Characteristics	MS *n* = 138	Without MS *n* = 357
Age (years)	53.3 ± 13.9	44.4 ± 14.8
Waist (cm)	108.1 ± 11.9	87.5 ± 12.8
Men	110.1 ± 9.7	92.4 ± 11.3
Women	106.3 ± 13.4	84.1 ± 12.6
BMI (kg/m²)	33.3 ± 5.4	26.2 ± 5.2
Men	32.2 ± 4.6	26.1 ± 4.5
Women	34.4 ± 5.9	26.2 ± 5.6
HOMA-IR	2.88 ± 1.97	1.11 ± 1.01
Men	3.02 ± 2.14	1.16 ± 1.28
Women	2.75 ± 1.80	1.07 ± 0.77
Systolic blood pressure (mmHg)	141.4 ± 14.5	124.2 ± 15.1
Diastolic blood pressure (mmHg)	87.5 ± 8.5	78.7 ± 9.2
Cholesterol (mmol/L)*	5.88 ± 1.18	5.63 ± 1.18
Triglycerides (mmol/L)	1.85 ± 0.89	1.10 ± 0.51
HDL-cholesterol (mmol/L) (men)	1.19 ± 0.36	1.48 ± 0.42
HDL-cholesterol (mmol/L) (women)	1.36 ± 0.36	1.74 ± 0.43
Fasting glucose (mmol/L)	6.14 ± 1.08	5.16 ± 0.46
2h glucose during OGTT (mmol/L)	7.22 ± 2.79	5.11 ± 1.81
Fasting insulin (mU/L)	10.44 ± 6.37	4.76 ± 4.20
Impaired fasting glucose^*¤*,$^	12 (9)	13 (4)
Impaired glucose tolerance^*¤*^	27 (20)	17 (5)
Diabetes^*¤*^	33 (24)	6 (2)

Data presented as mean ± SD, ^*¤*^data presented as *n* (%), *P* < 0.0001, ^$^ *P* = 0.04, *P = 0.03.

OGTT: oral glucose tolerance test.

**Table 3:** Comparison of premorbid^1^ and morbid^2^ subgroup in subjects with metabolic syndrome.

Characteristics	Premorbid MS subgroup *n* = 51	Morbid MS subgroup *n* = 87	*P* value
Age (years)	45.86 ± 14.6	57.72 ± 11.3	<0.0001
Waist (cm)	105.3 ± 12.7	109.7 ± 11.1	0.02
Men	106.6 ± 9.9	112.8 ± 8.8	0.008
Women	103.7 ± 15.8	107.5 ± 12.1	0.19
BMI (kg/m²)	32.2 ± 6.1	34.0 ± 25.1	0.03
Men	30.8 ± 4.6	33.2 ± 4.4	0.0495
Women	34.1 ± 7.4	34.6 ± 32.7	0.47
HOMA-IR	2.73 ± 1.8	2.97 ± 2.1	0.50
Men	2.87 ± 1.9	3.14 ± 2.3	0.65
Women	2.54 ± 1.7	2.84 ± 1.8	0.51
Systolic blood pressure (mmHg)	137.1 ± 14.0	143.9 ± 14.2	0.007
Diastolic blood pressure (mmHg)	87.2 ± 8.4	87.7 ± 8.7	0.72
Cholesterol (mmol/L)	6.0 ± 1.4	5.8 ± 1.1	0.85
Triglycerides (mmol/L)	2.03 ± 1.04	1.75 ± 0.77	0.22
HDL-cholesterol (mmol/L) (men)	1.15 ± 0.31	1.22 ± 0.40	0.46
HDL-cholesterol (mmol/L) (women)	1.26 ± 0.26	1.40 ± 0.40	0.14
Fasting glucose (mmol/L)	5.8 ± 0.7	6.3 ± 1.2	0.02
2h glucose during OGTT (mmol/L)	6.5 ± 2.2	7.7 ± 3.1	0.03
Fasting insulin (mU/L)	10.5 ± 6.5	10.4 ± 6.3	0.96
Impaired fasting glucose^*¤*^	6 (12)	6 (7)	0.4
Impaired glucose tolerance^*¤*^	9 (18)	18 (21)	0.8
Diabetes^*¤*^	5 (10)	28 (32)	0.006

Data presented as mean ± SD; ^*¤*^data presented as *n* (%); OGTT: oral glucose tolerance test; Subjects without^1^or with^2^ previously diagnosed and/or treated diabetes, dyslipidaemia, and hypertension.
